# Computational Advances in Cardiovascular Health

**DOI:** 10.1155/2019/2985984

**Published:** 2019-06-13

**Authors:** Dominique J. Monlezun, Francesco Nordio, Tianhua Niu

**Affiliations:** ^1^Department of Cardiology, University of Texas M.D. Anderson Cancer Center, Houston, USA; ^2^Department of Medicine, Brigham and Women's Hospital, Harvard University, Boston, USA; ^3^Department of Medicine, Tulane University School of Medicine, New Orleans, USA

Cardiovascular disease (CVD) is the world's top mortality cause, accounting for 1 in 3 deaths and 1 in 5 dollars of the American healthcare system. Patients deserve more effective, affordable, equitable care—and now—yet we in the clinical and research community struggle to accelerate the rate of reliable and replicable results to keep pace with this growing global epidemic. Computational advances in statistics and machine learning are increasingly bridging the informatics and physiological pipeline uniting precision medicine and population health, getting us thankfully closer to adequately addressing patient needs.

Through improved computational models, we could attain a better understanding of the physiologic (-omics, electrophysiology, solid mechanics, fluid dynamics), clinical (disease trajectories, pharmacology, comorbidity interaction), and population (social networks, social determinants, policies, inequities, and ecosystems of health) factors that help produce the complex phenomenon of CVD. Computational advances must simplify the CVD problem, so we can act, reassess, and act again on the problem through incremental research gains in a growing snowball fashion. Clinically, patients need such methodological improvements; financially, their medical costs are nonsustainable; and most important, ethically, our patients deserve faster, better, cheaper results from medical science. Health systems internationally are responding to (or forced to react to) these societal changes—patients are rightfully demanding better treatments, clinicians are expecting better data to guide their decisions, payers are requiring higher value care, and regulatory and funding bodies are mandating greater transparent and impact research. Yet where to begin, or go from here in CVD, is nearly as obtuse as the complexity of the above challenges we face as there are no widely accepted evidence-based standards in these computational advances, nor how to optimally apply them to reverse the CVD epidemic.

This special issue therefore aims at accelerating the dissemination of promising CVD computational advances. We received 15 submissions worldwide, and after performing rigorous and careful peer review, 6 of them were selected for this special issue. These resultant published studies provided novel computational, statistical, and machine learning approaches or clinical application to improve modelling for blood flow dynamics, solid mechanics, breath wave dynamics, and electrical cardiac rhythm for such patient populations as those affected by aortic aneurysm and dissection, atrial fibrillation, congestive heart failure, and arrhythmias.

One flow dynamic study conducted by J. Febina et al. created a 3D non-Newtonian mathematical model of pulsatile cardiac blood flow using CT data to map thoracic aortic aneurysm for computational fluid dynamics (CFD) to thus demonstrate wall shear stress (WSS) as a predictor for impending rupture, risk stratified throughout the cardiac cycle and stress conditions. This model notably included the vortex formation pattern and flow reversal (as in WSS) and therefore provided evidence for WSS nonoverly predicted through non-Newtonian pulsatile flow (similar to the natural physiology of the cardiac cycle of contraction and relaxation) but overpredicted when the natural flow pattern of laminar-turbulent-laminar is added to the non-Newtonian pattern. Newtonian fluid dynamics on the other hand were deployed in a different study performed by I. Avrahami and colleagues to assess carotid blood flow and wall dynamics in multiple bifurcation scenarios (normal, narrowed postoperatively with suture, and widened with high, medium, and lower flexibility patches) using fluid-structure interaction (FSI) numerical simulations. This was done to aid in the clinical decision about suture-based carotid endarterectomy versus patch-based angioplasty. The analysis suggested primary suture was superior to patch angioplasty by higher stress and high oscillatory shear index (OSI) along with lower time-averaged wall shear stress (TAWSS), with the high flexibility patch outperforming the lower flexibility patch. Image improvement was the focus of a third flow dynamic study conducted by Huang and co-workers. In this, an image time-domain integration modelled on blood flow periodicity was proposed to streamline improvements in image and noise suppression for digital subtraction angiography (DSA). In this model, postcontrast cardiac cycle images were synthesized for independent application or as a postprocessing noise suppression technique. This approach demonstrated good real-time performance and efficiency for enhancement and noise suppression in aortic dissection. Flow dynamics were included with solid mechanics in another paper by N. Kiefer et al., in which a global optimization algorithm was proposed and deployed in 17 critically ill atrial fibrillation (AF) patients to approximate diastolic stiffness (to aid in the diagnosis of left ventricular diastolic dysfunction (LVDD)) through a mathematical solution for the ill-posed nonlinear inverse problem of parameter estimation rooted in the physiological model of diastolic filling. This study provides the first known quantification of LV diastolic function using routine clinical data from critically ill patients with AF.

Breath wave dynamics were assessed in the study of T.-C. Fu et al. using an autoregressive (AR) mathematical model created to analyze breath-by-breath exercise test data to better analyze cardiopulmonary exercise testing (CPET) exercised-induced periodic breathing (PB) to prognosticate congestive heart failure (CHF). Hilbert–Huang transform (HHT) was utilized to decompose the AR model's breath-by-breath values into intrinsic mode functions (IMFs), mathematically representative of individual breath's physiologic oscillation in various frequencies. This model demonstrated predictive performance for CHF prognosis based on the third and fourth IMF components among 61 CHF patients, evidencing the biologic basis as a physiologic reserve indicated by ventilation and the above HHT approach to modelling CHF.

Finally, electrical cardiac rhythm was studied in another paper of S. M. Anwar et al. on dynamic and morphological electrocardiogram (ECG) features which were utilized in a novel approach for arrhythmia classification using discrete wavelet transform (DWT, dimensionality reduced by independent component analysis to minimize redundancy) for heart beats and Teager energy operator for nonlinear dynamic RR intervals. These features were then run through a threefold cross-validation neural network algorithm, which was then compared with the MIT-BIH databases for arrhythmia (13,724 beats) and supraventricular arrhythmia (22,151 beats). Accuracy for class- and subject-orientated schemes was improved to 99.75% and 99.84% using this unique approach. Machine learning methods, such as neural network applied in this paper, and other popular algorithms including support vector machine, k-nearest neighbor, decision tree, and random forest, are gaining attention in their applications for -omics research and precision medicine to augment translational clinical research, notably by providing the unique advantages of handling high-dimensional data more efficiently than traditional statistical strategies.

To the researchers who submitted these accepted manuscripts and those which were not, we are grateful for their shared commitment to improved scientific understanding and thus clinical care. We hope that, in some small way, the cardiovascular computational advances they contain may not only improve the patient care to which they apply but also accelerate the CVD research to which their methodologies can be more broadly adapted. Our patients and populations deserve better care. And so we offer this special issue as a humble step forward, believing in the promise of such computational advances to help speed up the journey for conquering CVD together as a global human family.

